# Open questions: knowing who’s who in multicellular animals is not always as simple as we imagine

**DOI:** 10.1186/s12915-018-0582-8

**Published:** 2018-10-15

**Authors:** Robin A. Weiss

**Affiliations:** Division of Infection & Immunity, University College London, Cruciform Building 1.3, Gower Street, London, WC1E 6BT UK

## Abstract

The ability of certain tumor cells of mammals and molluscs to spread from the original host to others reopens the question of distinguishing self from non-self. It is part of a wider phenomenon of cellular parasitism and cell chimerism including germ cells.

## What constitutes an individual organism?

Let us consider a multicellular animal—a human, for example. Each of us is a mobile ecosystem carrying a commensal microbiome including 1000s of species of bacteria outnumbering human cells by about 100:1 and a virome by about 10,000:1. But we assume that all our human cells are derived by cell division from a single fertilized egg. I shall discuss some exceptions, chimeric animals and the emergence of selfish cells that colonize new individuals, for which, from his studies of chimerism in the fruiting bodies of the cellular slime mold *Dictyostelium mucoroides*, Leo Buss coined the term somatic cell parasitism [1].

My interest in what constitutes an individual was first aroused by two books I read when I was a zoology undergraduate. In 1911, Julian Huxley wrote a slim volume called *The individual in the animal kingdom* and in 1958, Peter Medawar published *The uniqueness of the individual* (recently reissued by Scholar Select). Huxley and Medawar addressed the self/non-self paradigm of recognition and rejection of cells of a ‘foreign’ provenance. Huxley noted that when cells taken from sponges of two species are mixed together, they initially form one reaggregated organism but later separate to form two, one of each genotype. Clearly, some form of self-recognition operates even among the most primitive multicellular animals. Medawar investigated graft rejection in mice mediated by transplantation (histocompatibility) antigens that vary between individuals within a species. Although the nature of these molecules and the role of the dominant component, the major histocompatibility complex (MHC), in antigen presentation had yet to be elucidated, he and his colleagues deduced the basis of genetic individuality and demonstrated acquired immunological tolerance when mismatched cells were mixed early in development.

In 1983, my curiosity was further piqued by a paper from epidemiologists at the US National Cancer Institute suggesting that canine transmissible venereal tumor (CTVT) might serve as a model for Kaposi’s sarcoma in AIDS. CTVT was thought by some veterinarians to be a naturally transplantable tumor among outbred dogs and certain chromosome and genetic markers supported this hypothesis. However, oncologists well versed in transplant immunology regarded it with deep suspicion. When Yuan Chang and Pat Moore showed in 1994 that Kaposi’s sarcoma is actually caused by a novel herpesvirus, KSHV, I began to study KSHV, but the notion of transmissible tumors remained lodged in my mind. So, I later persuaded a young veterinarian, Claudio Murgia, to address the question using ‘forensic DNA’ markers of dogs. We were able to confirm that the transmissible agent of CTVT is indeed the tumor cell itself and that it has spread globally amongst dogs as a single somatic cell clone.

## Contagious cancers

To date, transmissible tumor cells have been identified in dogs, in the Tasmanian devil (*Sarcophilus harrisii*), and in several species of marine bivalve molluscs. Extensive phylogenetic studies on the canine and devil tumors led by Liz Murchison in Cambridge have provided much insight into naturally transplantable tumors. The canine transmissible venereal tumor has diversified genetically since it emerged in an ancient dog breed [2]. As its name implies, CTVT is spread mainly by sexual intercourse, which in canids can have a long post-coitus period when the male is unable to disengage from the female, thus facilitating exchange of cells. The devil facial tumor (DFT) is chiefly spread by biting between these aggressive marsupial carnivores; in fact, two tumors of distinct provenance, DFT-1 and DFT-2, have emerged in this species [3]. David Metzger in Steve Goff’s Laboratory at Columbia University has shown that several types of bivalve mollusc develop leukemia by acquiring tumor cells from the sea water through filter-feeding, in one case from a different host species [4].

While the emergence of transmissible tumors appears to be rare in the natural world, their discovery poses several questions.

First, how many more examples may come to light if we probe further in the animal kingdom? The cellular transmission of the bivalve tumors only came to light in recent years. There are filter feeders among several animal phyla that would merit investigation.

Second, how do the tumor cells evade host immune responses? Many mammalian tumors down-regulate expression of histocompatibility antigens, or express inhibitory cytokines such as TNFβ, and mutate genes conferring growth advantage, but few have developed the propensity to spread from host to host.

Third, how long can tumor cell parasites perpetuate themselves in their host population? CTVT is estimated to be 8,000–11,000 years old [2]. It was first investigated experimentally in 1876 by the Russian veterinarian Mstislav Novinsky, who showed that the tumor can be serially transplanted by inoculation into unrelated dogs; it can also be xenografted into other canid species. For many years around the turn of the twentieth century, CTVT was the major tumor used in allograft studies until the development of highly inbred rats and mice allowed transplantation of benign and malignant tissues in rodents.

For CTVT to be self-sustaining over countless generations, it would not pay to be too virulent. Female dogs may need to survive until the next estrus for the tumor cells to multiply and move on to the next host. Some cases of CTVT naturally regress after a period of rapid growth and, as Anton Sticker showed in 1906, recovered dogs are immune to re-inoculation. Regression can be induced by vincristine treatment, which Ari Fassati at UCL recently showed activates acute innate immune responses in the host stroma rather than the tumor cells, triggering immune cell infiltration and tumor rejection [5]. In contrast to CTVT, DFT in Tasmanian devils is a relentlessly progressive and fatal disease and the epidemic of the two tumors poses a threat to the survival of this endangered species [3]. DFT was first observed in 1996 and its origin probably dates in decades rather than millennia. Although the devil has suffered severe population bottlenecks and may be partially inbred, they reject skin transplants, suggesting that DFTs have lost or downregulated some transplantation antigens.

DFT-1 and DFT-2 probably have a neuro-endocrine origin [3]. CTVT was thought to be of ‘histiocytic’ origin, that is, from cells of the myelo-monocytic lineage, although its transcriptome indicates similarities to melanoma [5]. It is therefore possible that both canine and devil tumors have arisen from cells of neural crest ontology, a naturally migratory type of cell. During countless passages through different hosts, CTVT acquires new attributes. For example, host mitochondria, which presumably confer selective growth advantage, have invaded the CTVT lineage on at least five occasions [6]. Thus, while the tumor cell emerged as a transmissible parasite, host mitochondria have in turn colonized the tumor.

The leukemias of bivalve molluscs on the east and west coasts of the USA and Canada are spread by dissemination through seawater to distant sessile filter-feeders, providing a novel frisson to enjoying a clam bake on the beach. Several different species of clams, mussels and cockles have been found to harbor clonal transmissible tumors, and in one case at least, the malignant clone has taken up residency in a different species from that of its origin [4]. This phenomenon again raises the question of distinguishing self from non-self, especially where cells can freely diffuse or migrate in an aqueous environment. Might the emergence of transmissible tumors have led to past extinctions in vertebrate species, rather as DFT threatens devils today?

CTVT originated in a dog related to ancient Siberian breeds [2]. Ancient Siberian dogs also accompanied humans migrating across the Bering Strait. Genome analysis shows that they thrived in the Americas for ~ 8,000 years until an abrupt and terminal decline followed the introduction of modern dogs from Europe. In a commentary on a recent paper describing the genomes of pre-Columbian dogs, Goodman and Karlsson [7] postulate that CTVT may have been responsible for their virtual disappearance. If one of the European dogs carried CTVT, upon transfer it could well have been far more virulent in ‘native American’ dogs owing to fewer histocompatibility differences. However, CTVT has not threatened the survival of more distantly related canine populations so a partial immune response may confer a steady-state balance that benefits the host by modulating the growth of the parasite, thus promoting the survival of both. Could the cross-species transfer of tumors in clams [4] also benefit both tumor and host if it were less virulent in the foreign host?

## Invasion by normal cells

Transmissible tumors are the ultimate selfish parasites, but non-malignant cells can also colonize new individuals. This phenomenon has been studied most intensively in colonial ascidians by Irv Weissman [8] at Stanford and extended by Buki Rinkevich at the National Institute of Oceanography, Haifa. Ascidian protochordates form large colonies of ‘zooids’ where it may be difficult for biologists (and for somatic cells) to determine where one soma ends and another begins. Any cell lineage that had a slight selective advantage, even if it is not malignant, could migrate into other members of the colony. However, while chimeric zooids readily form by fusion at colonial borders, they tend to sort and separate over time, like Huxley’s sponges.

The ultimate sneaky way to propagate one’s own genes with minimal investment into body-building occurs when germ cells migrate into the soma of a neighboring zooid [8]. I wonder whether this cuckoo-like phenomenon of parasitic germ cells might occur in other colonial animals, such as bryozoans or among colonial *Cnidaria* (Coelenterates), the sessile corals and the floating Portuguese man-o-war?

## Self and non-self

Most multicellular animals have rudimentary identification systems for recognizing cells of different genetic origin, as mentioned already for sponges, cellular slime molds and ascidians. Indeed, multiple mating types in sexual eukaryotes such as fungi and *Tetrahymena* represent analogous recognition systems of self from non-self, in this case to block self-fertilization. But the most sophisticated recognition system is that of within-species MHC polymorphism that evolved concomitantly with adaptive immunity in the vertebrate lineage.

In the Division of Infection & Immunity, which is my emeritus home, we teach students that adaptive immunity and the MHC system serve to protect the host from pathogenic infections. Quite so, they do, since more frequent infections and increased virulence are evident in immunodeficient individuals, and past epidemics have influenced MHC allele frequencies in populations. However, like Buss [1], I would wager that protection against the emergence of parasitic cells has also been an important driver in the evolution of self/non-self recognition, including the polymorphic histocompatibility systems of vertebrates. MHC protects us from potentially contagious cancer cells.

## Chimerism

In mammals, adaptive cellular immunity develops late in gestation or after birth. If exchange of blood cells or stem cells occurs between fetuses in utero, the resulting individuals do not reject each other’s cells or tissues and form chimeras with long-lasting cells derived from both individuals. This was first recognized by Ray Owen in 1945 in ‘freemartin’ twin cattle and led Medawar and colleagues towards the discovery of acquired immunological tolerance. We now know that some New World primates such as marmoset twins naturally exchange fetal cells in fused placentas, leading to sibling chimerism of many tissues, including germ cells [9].

Returning to the theme of tumor cells, humans can be prenatally seeded with allogeneic leukemic cell precursors from a twin via placental anastomoses or exposure to the maternal circulation [10]. Of course, pregnant females tolerate their ‘engrafted’ fetuses, to a point. Moreover, human malignant choriocarcinoma is another example of a transmissible tumor, albeit to only one recipient—derived from the placental trophoblast and invading the mother. Perhaps its non-self nature explains why choriocarcinoma is usually curable following chemotherapy, like CTVT.

Overall, the concept of individuality and the phenomenon of cellular transmission still present us with open questions.
